# Sequential Tool Use in Great Apes

**DOI:** 10.1371/journal.pone.0052074

**Published:** 2012-12-26

**Authors:** Gema Martin-Ordas, Lena Schumacher, Josep Call

**Affiliations:** 1 Max Planck Institute for Evolutionary Anthropology, Leipzig, Germany; 2 Center on Autobiographical Memory Research, Aarhus, Denmark; Université Paris 13, France

## Abstract

Sequential tool use is defined as using a tool to obtain another non-food object which subsequently itself will serve as a tool to act upon a further (sub)goal. Previous studies have shown that birds and great apes succeed in such tasks. However, the inclusion of a training phase for each of the sequential steps and the low cost associated with retrieving the longest tools limits the scope of the conclusions. The goal of the experiments presented here was, first to replicate a previous study on sequential tool use conducted on New Caledonian crows and, second, extend this work by increasing the cost of retrieving a tool in order to test tool selectivity of apes. In Experiment 1, we presented chimpanzees, orangutans and bonobos with an out-of-reach reward, two tools that were available but too short to reach the food and four out-of-reach tools differing in functionality. Similar to crows, apes spontaneously used up to 3 tools in sequence to get the reward and also showed a strong preference for the longest out-of reach tool independently of the distance of the food. In Experiment 2, we increased the cost of reaching for the longest out-of reach tool. Now apes used up to 5 tools in sequence to get the reward and became more selective in their choice of the longest tool as the costs of its retrieval increased. The findings of the studies presented here contribute to the growing body of comparative research on tool use.

## Introduction

Several non-human animal species are capable of using tools [2], [3]. However, capuchin monkeys, apes, and corvids are the species that have produced the most impressive examples [4], [5]. Among those are cases of sequential tool use in which a tool is used to obtain another tool, which subsequently will serve to obtain an out-of-reach goal (e.g., food). Nonetheless, instances of sequential tool use among non-human animals are rather scarce. Bird & Emery [6] have argued that the difficulty in sequential tool use stems from three problems: First, the subject must recognize that one tool can be used on another or on nonfood items. Second, the subject must resist the immediate motivation to use the tool to attempt to access the food directly, and third, the individual must be capable of hierarchically organized behavior.

In the laboratory, one of the most common tasks to test sequential tool use consists of presenting subjects with a reward that is out of reach, a readily available tool that is not long enough to reach for the reward but long enough to reach for another tool, which can be used to reach for the reward. Subjects have to use the tools sequentially by first using the shorter tool to retrieve the longer tool and secondly using the latter to reach for the food. Spontaneous use of up to two tools in sequence has been reported in chimpanzees [7], gorillas and orangutans [8] and capuchin monkeys [9]. Macaques [10] and cotton-top tamarins [11] can also use tool in sequence after receiving some training. In fact, training was instrumental in one of the most impressive instances of sequential tool use ever recorded [12]. The chimpanzee Julia proved capable of using up to five tools in sequence. The difficulty in the task resided in the fact that she had to look at a transparent, locked box to determine what kind of key was needed to open it, and then find that key in another transparent locked box. This second box in turn also required a key that Julia had to find in still another transparent locked box and so on up to five boxes, all presented simultaneously. It is unclear whether Julia would have succeeded without the benefit of the various pre-training phases that she received.

More recent investigations of sequential tool use in corvids have shown that this ability is not only exclusive to primates. Non-tool using rooks (*Corvus frugilegus*) have been reported to use tools sequentially by spontaneously dropping a large stone into a container to release a small stone, which was then used to acquire food [6]. In another study, New Caledonian crows were reported to use an immediately available short stick to reach for an out-of-reach long stick (placed in a box), and subsequently use the long tool to reach for the reward in a vertical tube [13], [14].

Wimpenny, Weir, Clayton, Rutz and Kacelnik [1] have also reported that New Caledonian crows use tools in a sequence using a different experimental setup. In this study, crows were presented with an out-of-reach reward, two tools that were available but too short to reach the food and four out-of-reach tools differing in functionality. The distance of the food and/or which tools were required to get it defined the different experimental conditions. Therefore, the position of the food reward and/or tools dictated what sequence of behavioral actions was required for a successful completion of the task. Wimpenny et al. [1] found that crows were able to use up to three tools in sequence in order to get the reward. One of the successful subjects did so even when he did not receive any pre-training with the elements of the task. However, subjects’ performance was not perfect. In fact, subjects sometimes used small tools to fish for longer ones when there was no ultimate food reward present.

However, all the above-mentioned studies have some methodological limitations that prevent us from drawing unambiguous conclusions regarding subjects’ performances in the sequential tool-use tasks (see Wimpenny et al., [1]). First, in most of the studies the out-of-reach tool was positioned in close proximity to the food reward or between the subject and the reward [10], [11], [15], [16]. Therefore, using this set-up does not rule out a potential retrieval of the inaccessible tool by misdirecting the immediately available tool towards the out of reach tool and, thus, retrieving the latter by chance. Second, in some studies, subjects received training on the basic elements of the tasks (i.e. reaching for food with a tool and/or interacting with the constructs later containing the inaccessible tool(s)) before being presented with the actual test [e.g. 8, 10, 13, 14, 15]. Hence, such procedure could potentially have enabled them to solve the tasks by simply chaining the crucial elements which had become secondary reinforcers. Epstein, Kirshnit, Lanza, Rubin [17] suggested that linking the previously learned behaviors into a novel sequence was the product of simple, associative learning mechanisms such as competition between behavioral repertories, automatic chaining, and functional generalization. Nevertheless, linking might also be dependent on the ability of an animal to organize learned behaviors hierarchically into behavioral chains with goals and sub-goals, although, to date, there is no conclusive evidence for this [18], [19].

Third, some studies have presented the animals with only the potential correct tools to solve the problem [10], [11], [13], [14]. Therefore, it is not surprising that the animals tried to use those tools in some way, especially if the tools were sticks for which the animal had a natural or learned predisposition to manipulate. Thus, presenting subjects with several tool options might be more informative because they are required not only to use tool in sequence but also to select which tools are necessary to solve the task. Wimpenny et al. [1] found that some of the inexperienced New Caledonian crows they tested did not always chose the correct stick under those conditions.

Finally, the studies on sequential tool use have not controlled for the cost of retrieving the out-of-reach tool/s. For instance, Wimpenny et al. [1] concluded that crows did not take into account the distance at which the food was placed in the tube because subjects tended to always retrieve the longest out-of-reach tool. However, while the cost of retrieving the longest out-of-reach tool was not very high (i.e. except for one of the conditions, all tools were evenly aligned and, therefore, equally accessible), the benefits of using the longest tool were always extremely high (i.e. the longest out-of-reach tool was the only tool that allowed subjects to succeed in all the experimental trials).

In the current study we investigated sequential tool use in chimpanzees (*Pan troglodytes*), orangutans (*Pongo abelii*) and bonobos (*Pan paniscus*) using the same setup that Wimpenny et al. [1] used to test New Caledonian crows. In particular, subjects faced an out-of-reach piece of food, two readily available tools (only one of which was sufficiently long to retrieve either food or further tools) and four out-of-reach tools. The food reward was placed on the opposite side from the out-of-reach tools so that the direct visual comparison of depth of the reward and lengths of tools was impossible. In different conditions we varied the distance of the food and/or which tools were required to get it. What sequence of behavior was required to solve the different conditions depended on the position of the food reward and/or tools. In order to solve the task, subjects had to use the longer stick readily available to reach for a longer out-of-reach tool, which allowed them to retrieve that reward. These sequences of actions were necessary for all the sequential trials except for the one in which the food was placed the closest to the subjects (i.e. Primary). Then, the longer of the two readily available tools sufficed to retrieve the reward.

We adopted Wimpenny’s et al. [1] design for two reasons. First, it allowed us to directly compare the performance of three great ape species with New Caledonian crows, thus fostering direct comparisons both inside and outside the great ape clade. The comparison between apes and corvids, especially those that typically use tools, is particularly appealing in light of the idea that these two taxonomic groups have undergone convergent cognitive evolution [20]. Second, Wimpenny’s et al. [1] design controlled for some of the methodological limitations of previous studies. In particular, the food was not close to the tools, and its distance could not be directly compared to the length of the tools. Unlike Wimpenny et al. [1], however, we did not train the apes on any task prerequisites although all apes had experience using tools and some had been tested in a sequential tool-use task (see Methods). Additionally, we increased the costs of reaching for the longest out-of-reach tools in Experiment 2. Such variations would allow us to draw more precise conclusions about which elements of the task subjects took into account when retrieving an out-of-reach tool (i.e. whether subjects took into account the distance at which the food was placed in order to select the tool of the appropriate length).

### Experiment 1

In this experiment we closely followed Wimpenny et al.’s [1] setup with the New Caledonian crows. Subjects were presented with four out-of-reach tools, which could be extracted by using an immediately available short stick. The distance at which the food was placed on the platform, defined the number of tools necessary to obtain the reward. Therefore, depending on the food distance, subjects were required to use from 1 to up 3 tools. It is important to note, though, that in contrast to the study with the crows, our subjects did not receive any pre-testing experience.

## Materials and Methods

### Subjects

Eight chimpanzees (*Pan troglodytes*), three bonobos (*Pan paniscus*) and four orangutans (*Pongo abelii*) housed at the Wolfgang Köhler Primate Research Center (WKPRC) in the Leipzig Zoo participated in this experiment. There were 9 females and 6 males ranging from 10 to 29 years of age (see Table 1). All subjects had participated in a variety of cognitive tests, some of which included tasks involving sequential-tool use (see Table 1). Groups of apes were housed in semi-natural indoor and outdoor enclosures with regular feedings, daily enrichment and water ad lib. Subjects voluntarily participated in the study and were never food or water deprived. Research was conducted in the sleeping rooms. No medical, toxicological or neurobiological research of any kind is conducted at the WKPRC. Research was non-invasive and strictly adhered to the legal requirements of Germany. The study was ethically approved by an internal committee at the Max Planck Institute for Evolutionary Anthropology (the joint ethics committee of the MPI-EVA and the Zoo Leipzig). Animal husbandry and research comply with the “EAZA Minimum Standards for the Accommodation and Care of Animals in Zoos and Aquaria”, the “WAZA Ethical Guidelines for the Conduct of Research on Animals by Zoos and Aquariums” and the “Guidelines for the Treatment of Animals in Behavioral Research and Teaching” of the Association for the Study of Animal Behavior (ASAB). IRB approval was not necessary because no special permission for the use of animals in purely behavioral or observational studies is required in Germany.

**Table 1:** Name, gender, age, rearing history, order in which experiments were conducted and objects used for Non-tools condition (* indicates previous subjects’ experience in sequential tool use tasks).

Subject	Gender	Age (years)	Rearing history	Order	Objects
Chimpanzee					
*Frodo*	M	17	Mother raised	2, 1	Bricks
*Alex*	M	10	Mother raised	1,2	Bricks
*Lome*	M	10	Mother raised	1,2	Corks
*Jahaga*	F	18	Mother raised	2,1	Corks
*Fifi*	F	18	Mother raised	1,2	Bricks
*Sandra*	F	18	Mother raised	1,2	Bricks
*Pia*	F	12	Mother raised	2,1	Corks
*Alexandra*	F	12	Nursery raised	2,1	Corks
Bonobo					
*Joey*	M	28	Nursery raised	1,2	Corks
*Kuno*	M	14	Nursery raised	1,2	Bricks
*Yasa*	F	13	Unknown	1,2	Bricks
Orangutan					
*Bimbo* *	M	29	Nursery raised	1,2	Bricks
*Dokana* *	F	21	Mother raised	1,2	Corks
*Padana*	F	12	Mother raised	1,2	Corks
*Pini^1^* *	F	22	Mother raised	1,2	Bricks

* Mulcahy, Call & Dunbar (2005)

### Apparatus

**Figure 1:**
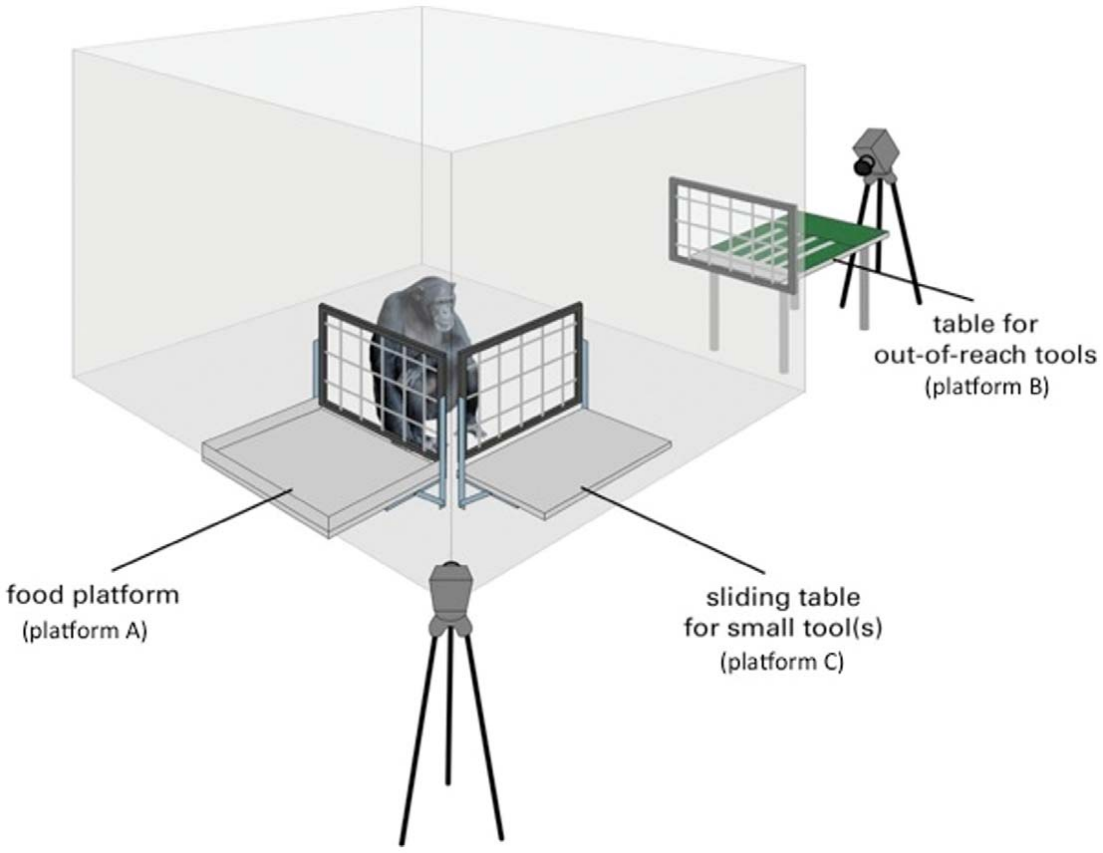
Experimental set-up for Experiment 1 and Experiment 2.

The test apparatus consisted of three platforms (see Figure 1): a food-platform (platform A: 79.5 cm width×55 cm length) where we placed the food, a sliding table (platform C: 78 cm width×37 cm length) where we placed the two within-reach tools, and a tools-platform (platform B: 65 cm width×104 cm length×51 cm height) where we placed the out-of-reach tools. Fixed to the surface of the latter, at a distance of 14 cm from the subjects, there were five equally sized parts of green-colored plastic boards (12 cm width×90 cm length and 3 mm thick). Between these boards we built four channels of approximately 1.3 cm width each, placed at 12 cm from each other, where we placed the tools.

Tools were wooden sticks with square cross section of 10 mm diameter. The immediately available tools or within-reach tools had a length of 10 cm and 20 cm, and the out-of-reach tools were 25 cm, 35 cm, 45 cm and 55 cm in length. The out-of-reach tools were spatially and visually separated from the reward (see Figure 1). We used banana slices as rewards. Corks (4.5 cm long and of 1.3 cm diameter) and wooden ‘bricks’ (3 cm×3 cm×2 cm) were used as no-tool objects.

### Procedure

Subjects were individually tested in their indoor cages after being separated from their groupmates. Young infants stayed with their mothers while the test took place. The experimenter (E) first placed the food on the platform A, then she placed the four out-of-reach tools on platform B and finally the two within-reach tools on platform C (Fig. 1). Before placing the out-of-reach tools, for each tool E showed its full length to the ape by holding crossways for 5 seconds. Note that platform B was positioned so that subjects were facing away from the platform A when retrieving the tools from the platform B (see Figure 1). A trial began when E pushed platform C towards the mesh panel and ended when either subjects obtained the food, they removed all tool/objects (in conditions No-food/No-tools), or after a maximum duration of 5 minutes. If subjects were still trying to get the tools or food after 5 minutes, the trial continued until they either obtained the food or they stopped reaching for more than 1 min.

There were different types of trials defined by the distance at which the food was placed (d1, d2, d3; see Table 2) and the number of tools required for retrieving the reward (see Tables 3). All subjects received three types of trials (Table 3): sequential (experimental) trials (Primary, Secondary-Any, Secondary-Long and Tertiary), no-goal (control) trials (‘No-food’ and ‘No- Tools’) and Length-only (control) trials (d1, d2, d3). In the sequential trials, the depth of the food and distance of the tools from the mesh dictated the sequence of behavior necessary to retrieve the food, with the most demanding condition being the Tertiary because it required the use of 3 tools in sequence (see Table 3 for a summary of all conditions). The use of tools that differed in functionality allowed us to investigate if apes were able to take into account the relevant features of the problem: distance at which the food was placed, the length of the tool required to retrieve the food, and number of tools necessary to obtain the reward.

**Table 2:** Distances (in cm.) at which the food was placed in Experiment 1 and Experiment 2 for each species.

	Experiment 1				Experiment 2			
	d1	d2	d3	No-tools	d1	d2	d3	d4
**Chimpanzees**	14	19	50	25.5	14	19	40	50
**Bonobos**	14	19	50	25.5	14	19	40	50
**Orangutans**	17	24.02	50	31	17	24.02	40	50

**Table 3:** Description of sequential and length-only trials for Experiment 1.

Condition	Conditiontype	Phase	Foodposition	Trial description	Most task-sensitivebehavior for success
**Secondary-any**	Sequential	1	d2	The four out-of-reach tools are placed flush with each other on the table. Food is within reach of any out-of-reach tool.	Get any out-of-reach tool with the 20 cm tool and use the extracted tool to reach for the food.
**Secondary-long**	Sequential	1	d3	The four out-of-reach tools are placed flush with each other on the table. Food is only reachable with the longest out-of-reach tool.	Extract the longest out-of-reach tool (55 cm) with the 20 cm tool and use it to reach for the food.
**Tertiary**	Sequential	1	d3	The 25 cm, 35 cm and 45 cm tools are placed flush with each other on the table, but the longest tool (55 cm) is displaced backwards by some distance. Food is only reachable with the longest out-of-reach tool, which is only reachable with the 45 cm out-of-reach tool.	Get the 45 cm out-of-reach tool with the 20 cm tool. Then use the 45 cm tool to reach for the longest out-of-reach tool (55 cm). Use the 55 cm tool to reach for the reward.
**Primary**	Control	1	d1	Food is within reach of the longer tool subjects are provided with (20 cm).	Reach for the food with the 20 cm. Do not probe for any out-of-reach tool.
**No-food**	Control	1	not applicable	No food is placed on the platform but tools are placed as usual.	Do not probe for anything.
**No-tools**	Control	1	intermediate	Food is placed at an intermediate depth. Instead of the out-of-reach tools objects (wooden bricks or corks) are placed on the table.	Do not probe for anything.
**Length-only** **(d1/d2/d3)**	Control	2	d1, d2, d3	These three types of length-only trials correspond to the primary, secondary-any and secondary-long/tertiary trials. In contrast, the four tools are now placed on a tray within reach of the subjects.	Length-only (d1): reach for food with the 20 cm tool. Length-only (d2): Get any tool from the tray and then reach for the reward. Length-only (d3): Get the longest tool (55 cm) from the tray and then reach for the food.

In No-food trials, no reward was present. The purpose of these trials was to test firstly whether tools would still be extracted (in which case the tools themselves may be reinforcing), and secondly, whether subjects would probe the empty food-platform (which would indicate that the action of probing was relatively inflexible). In No-tools trials the tools were swapped for non-tool objects (wooden bricks or corks) and food was placed at an intermediate depth (see Table 2). The purpose of these trials was to see if subjects would probe for these objects, and if they retrieved them from the platform B, whether or not they would then use them in platform A.

In the Length-only control trials, the procedure was the same as the sequential trials with the important difference that now the out-of-reach tools were placed within subjects’ reach. Here, E placed the out-of-reach tools on the separate tray (50 cm×50 cm) 4 cm away from the mesh. Similar to the sequential trials, subjects were presented with the two within-reach tools. These trials were carried out to determine whether sequential tool use may have imposed additional cognitive demands that may have hampered tool selection. Following Wimpenny et al. [1]’s logic, if apes chose the wrong tool more often in the sequential trials than in the Length-only trials, this may indicate that the former involved higher cognitive demands than the latter.

Subjects received nine sessions of six trials each (54 trials in total). Only sequential trials and no-goal control trials were included in these nine sessions. Each type of sequential and no-goal control trials was randomly assigned within one session and each type of trial was presented only once in each session. The position of the out-of-reach tools and within-reach tools was counterbalanced across trials. For the non-tool conditions, 50% of the subjects were presented with the bricks as non-tool objects and the other 50% with the corks. Following the completion of these nine sessions, all subjects received an additional 30 ‘Length-only’ control trials distributed in 5 sessions with 6 trials each. Subjects received ten intermixed trials of each of three food depths: d1, d2 and d3 cm (each type of trial was presented twice in each session). All subjects received first Experiment 1 followed by Experiment 2. However, in order to rule out order effects, 50% of the chimpanzees were presented first with Experiment 1 and then Experiment 2 and for the other 50% received the reversed order.

### Data Scoring and Analysis

We videotaped all trials. For each trial we scored whether subjects retrieved the food (i.e. correct responses), which within-reach tool they retrieved, where they first probed (platform with the food or platform with the out-of-reach tools), which out-of-reach tool they retrieved first, and which out-of-reach tool they used first. A second independent observer scored a randomly selected sample of 20% of trials to assess inter-observer reliability, which was excellent for all the variables (retrieved food: Cohen’s k = 0.99; within-reach tools: Cohen’s k = 0.97; first probing: Cohen’s k = 0.97; first out-reach tool taken: Cohen’s k = 0.97; first-out-of-reach tool used: Cohen’s k = 0.92). Additionally, in the correct trials (i.e. subjects obtained the reward), we scored whether their correct performances were “perfect” (i.e. take the appropriate tool and use it) or whether it contained errors. A correct response was scored as “perfect” if it was sensitive to the demands of the tasks; for example, in the Primary condition the longest within-reach tool was long enough to retrieve the reward, therefore if apes used such tool rather than any of the out-of-reach tools, their response was scored as “perfect” (see Table 3 for the expected perfect responses in relation to the food location and length of the tools). We scored three different errors depending on the tools first taken and the tools first used: (a) subject takes the incorrect tool and uses it, (b) subject takes first the incorrect tool, then takes and uses the correct one, and (c) subject takes the correct tool, then takes the incorrect but uses the incorrect one.

We calculated the percentage of trials in which subjects obtained the reward (i.e. overall success). We used non-parametric tests because the data was not normally distributed. We used Friedman tests to analyze subjects’ success in the sequential trials and Length-only trials. Friedman tests were also used to investigate differences in first probing behaviors and first tools used. We also used Friedman tests to analyse subjects’ performance in the no-goal trials. Since Wimpenny et al. [1] examined crows’ behavior in these trials by comparing their performance in these control trials with their performance in the Secondary-any trial (because it was the simplest sequential trial), we did the same. Wilcoxon tests were run for post-hoc comparisons and to assess whether subjects performed above chance levels. We used the Kruskal-Wallis test to investigate species differences. Mann-Whitney-U test was used to analyze whether order in which the two experiments were presented had any effect in chimpanzees’ performance. Exact p values were calculated in all cases. All tests were two-tailed.

## Results

Since 50% of the chimpanzees were presented first with Experiment 1 and the other 50% with Experiment 2, we compared both groups’ performance in the sequential trials to check whether order of presentation had any significant effect on their success rates. A Mann-Whitney test revealed that success did not differ between the two groups of chimpanzees (Secondary-any: *U* = 4.000, *p* = 0.429; Secondary-long: *U* = 2.000, *p* = 0.143; Tertiary: *U* = 2.500, *p* = 0.143; Primary: *U* = 8.000, *p* = 1.000; *N* = 8 for all cases). Therefore, we pooled the data from the two groups for subsequent analyses.

### Sequential Trials

#### Success

**Figure 2:**
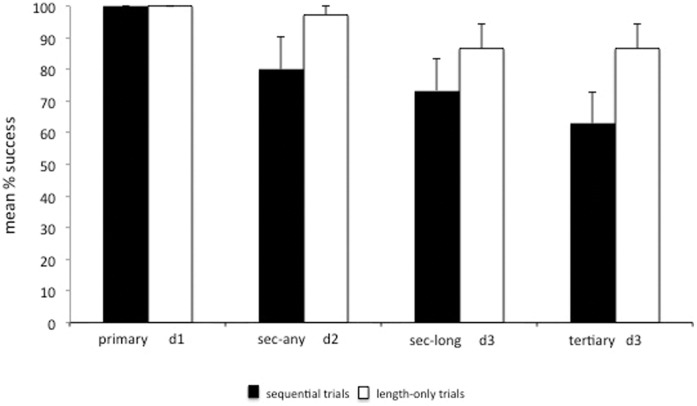
Mean % correct trials in the sequential and length-only trials (Experiment 1) [Error bars represent the standard error of mean].

We found that subjects’ overall success differed among the 4 types of sequential trials (Friedman test: χ^2^ = 25.67, df = 3, p<0.001). Whereas all subjects obtained food in all the Primary trials, their success rates in the other conditions decreased as the task complexity increased (see Figure 2). In fact, post-hoc tests showed that subjects performed significantly better in the Secondary-any (Wilcoxon test: T = 0.00, p = 0.004, n = 9) and Secondary-long conditions (T = 0.00, p = 0.008, n = 8) than in the Tertiary conditions. Subjects also performed better in the Primary condition than in the Secondary-long (Wilcoxon test: T = 0.00, p = 0.008, n = 8) and Tertiary conditions (T = 0.00, p = 0.001, n = 11). We did not find species differences for any of the sequential trials (Kruskal-Wallis test: Primary: χ*^2^* = 0.000, df = 2, *p* = 1.000; Secondary-any: χ*^2^* = 0.126, df = 2, *p* = 0.877; Secondary-long: χ*^2^* = 2.934, df = 2, *p* = 0.241; Tertiary: χ*^2^* = 3.671, df = 2, *p* = 0.171; *N* = 15 in all cases). Subjects solved the Secondary-any (*χ^2^* = 6.23, df = 1, p = 0.013, 11/13 correct) and Secondary-long conditions (*χ^2^* = 8.33, df = 1, p = 0.004, 11/12 correct) significantly above chance in the first trial. This was not the case for the Tertiary condition (*χ^2^* = 1.33, df = 1, p = 0.248, 4/12 correct) (see Table 4 for individual performances).

**Table 4:** First session in which individuals solved each experimental condition in Experiment 1 and individual performances (%) in the sequential and length-only trials.

Subject		Session			Sequential trials			Length-	only	trials
	sec-any	sec-long	tertiary	primary	sec-any	sec-long	tertiary	d1	d2	d3
Joey	-	-	-	100	0	0	0	100	100	60
Kuno	1	1	5	100	100	77.78	55.56	100	100	100
Yasa	1	1	4	100	100	77.78	77.78	100	100	100
Dokana	1	1	2	100	100	100	88.89	100	100	100
Padana	1	1	4	100	100	100	66.67	100	100	100
Pini	1	1	4	100	100	88.89	66.67	100	100	100
Bimbo	6	-	-	100	11.11	0	0	100	100	0
Alex	-	-	-	100	0	0	0	100	60	0
Fifi	1	1	1	100	100	100	100	100	100	100
Lome	2	2	3	100	88.89	88.89	77.78	100	100	100
Sandra	1	1	2	100	100	66.67	33.33	100	100	100
Alexandra	1	1	1	100	100	100	100	100	100	100
Jahaga	1	1	1	100	100	100	100	100	100	100
Frodo	1	1	2	100	100	100	88.89	100	100	100
Pia	1	1	1	100	100	100	100	100	100	100
**Median**	**1**	**1**	**2**							

#### Out-of-reach-tools or food

Except for 3 subjects (Alex, Bimbo and Joey), all apes reached for the out-of-reach tools in the sequential trials (see Table 4 for individual performances in each type of trial). Apes chose significantly above chance the 20 cm within-reach tool (as opposed to the 10 cm tool) in 91.35% of the trials (Wilcoxon test: T = 0.00, p<0.001, n = 15). When we analyzed if subjects used the 20 cm tool to reach for food or to reach for the out-of-reach tools, we found significant differences in their performance among the 4 different types of trials (Friedman test: χ^2^ = 26.56, df = 3, p<0.001; mean % (SEM): Primary = 44.44 (10.45), Secondary-any = 62.21 (9.25), Secondary-long = 82.96 (7.26), Tertiary = 77.77 (9.26)). Subjects reached for the out-of-reach tools significantly more often in the Secondary-long and Tertiary than in the Primary trials (Wilcoxon test: T_SecLong_ = 0.00, p<0.001, n = 13; Wilcoxon test: T_Tertiary_ = 0.00, p = 0.001, n = 11) and Secondary-any trials (Wilcoxon test: T_SecLong_ = 6.50, p = 0.003, n = 12; Wilcoxon test: T_Tertiary_ = 3.50, p = 0.021, n = 9).

#### First out-of-reach tool used

**Figure 3:**
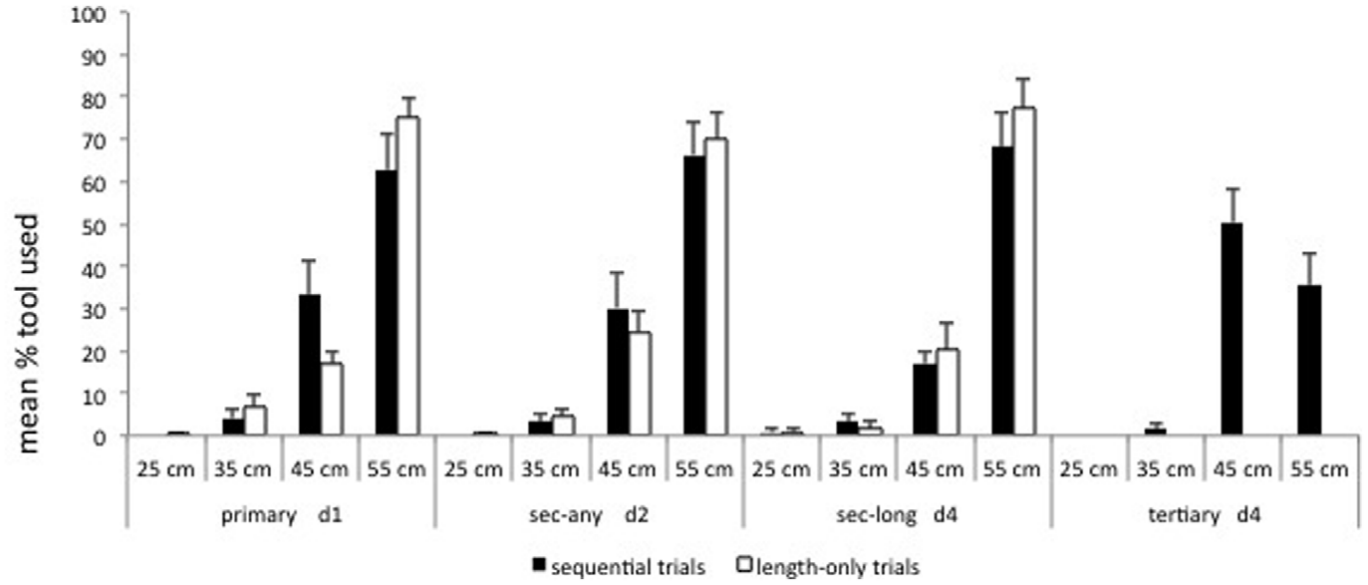
Mean % of first out-of-reach tool used in the sequential and length-only control trials (Experiment 1) [Error bars represent the standard error of mean].

We found significant differences among the 4 out-of-reach tools that apes used to get the food in the Primary condition (Friedman test: χ^2^ = 25.93, df = 3, p<0.001); Secondary-any condition (Friedman test: χ^2^ = 29.06, df = 3, p<0.001); Secondary-long condition (Friedman test: χ^2^ = 31.33, df = 3, p<0.001) and Tertiary condition (Friedman test: χ^2^ = 29.68, df = 3, p<0.001) (see Figure 3). Subjects showed a preference for using the longest out of reach tool in all conditions (Wilcoxon test: Primary T = 1.00, p = 0.016, n = 8; Secondary-any: T = 8.50, p = 0.007, n = 13; Secondary-long: T = 1.00, p = 0.001, n = 12) except for the Tertiary condition in which they selected the 45- and 55-cm tool equally (Wilcoxon test: T = 28.00, p = 0.682, n = 11) (see Figure 3). A comparison of the average length of the tool selected in each condition revealed significant differences in the length of tool used (Friedman test: χ^2^ = 10.825, df = 3, p = 0.010; mean cm. (SEM): Primary = 43.03 (5.35), Secondary-any = 51.28 (0.75), Secondary-long = 51.54 (0.90), Tertiary = 44.97 (3.81)), although such differences were not related to the distance of the reward (see Figure 3).

#### Errors

To further investigate subjects’ performance, we examined the errors that they made in the sequential trials in which they successfully retrieved the reward. Results showed that in the Secondary-any (Wilcoxon test: T = 0.00, p<0.001, n = 13; mean % (SEM): perfect = 84.39 (3.49); errors = 15.61 (3.49)) and Secondary-long trials (Wilcoxon test: T = 11.00, p = 0.047, n = 11; mean % (SEM): perfect = 62.19 (5.05); errors = 37.81 (5.50)) subjects’ perfect responses were more frequent than responses containing errors. We did not find significant differences between perfect performances and responses containing errors in the Primary trials (Wilcoxon test: T = 48.50, p = 0.529, n = 15; mean % (SEM): perfect = 54.81 (10.59); errors = 45.19 (10.78)). In the Tertiary trials, perfect performances were significantly lower than performances with errors (Wilcoxon test: T = 9.00, p = 0.016, n = 12; mean % (SEM): perfect = 33.32 (5.82); errors = 66.68 (5.99)).

### Length-only Trials

#### Success

Results showed no significant differences in subjects’ overall success across the 3 different food depths (Friedman test: χ^2^ = 5.60, df = 2, p = 0.111; Figure 2). When we compared subjects’ performance in the sequential trials with their corresponding trials in the Length-only condition (see Figure 2), we found that subjects’ overall success was significantly better for the d3 condition than the Secondary-long condition (Wilcoxon test: T = 0.00, p = 0.016, n = 7) and Tertiary (Wilcoxon test: T = 0.00, p = 0.002, n = 10).

#### First (out-of-reach) tool used

Subjects only took the 20 cm within reach tool in 2.67% of the trials and they never took the 10 cm within-reach tool. We found significant differences among the 4 tools that apes used to get the food in the d1 condition (Friedman test: χ^2^ = 40.42, df = 3, p<0.001); d2 (Friedman test: χ^2^ = 28.15, df = 3, p<0.001) and d3 condition (Friedman test: χ^2^ = 32.69, df = 3, p<0.001). In all conditions subjects showed a clear preference for using the longest tool (Wilcoxon test: d1 T = 0.00, p<0.001, n = 15; d2: T = 5.50, p = 0.001, n = 14; d3: T = 13.00, p = 0.005, n = 15) (see Figure 3). A comparison of the average length of the tool selected in each condition revealed no significant increase in tool length as a function of the distance to the reward (Friedman test: χ^2^ = 1.84, df = 3, p = 0.429; mean cm. (SEM): d1 = 50.73 (0.66), d2 = 50.84 (0.82), d3 = 51.71 (0.67), d4 = 53.07 (0.36)).

#### Errors

We examined whether the correct responses contained errors and we compared perfect performances between sequential and Length-only trials. We found significant differences between perfect performances and responses containing errors in d1 (Wilcoxon test: T = 0.00, p<0.001, n = 15; mean % (SEM): perfect = 2.00 (1.44); errors = 98.00 (1.44)) and d2 trials (Wilcoxon test: T = 0.00, p<0.001, n = 14; mean % (SEM): perfect = 98.00 (1.06); errors = 2.00 (1.06)) but not in d3 trials (Wilcoxon test: T = 20.50, p = 0.167, n = 12; mean % (SEM): perfect = 61.19 (6.97); errors = 38.80 (6.97)).

We found that subjects’ perfect performance did not occur more often in d3 than Secondary long (Wilcoxon test: T = 28.50, p = 0.724, n = 11; mean % (SEM): Secondary-long = 62.19 (5.05); d3 = 61.19 (6.97)). Perfect performances occurred more often in Primary trials than in d1 trials (Wilcoxon test: T = 0.00, p<0.001, n = 12; mean % (SEM): Primary = 54.81 (10.59); d1 = 2 (1.44)), in d2 than Secondary-any trials (Wilcoxon test: T = 1, p = 0.004, n = 10; mean % (SEM): Secondary-any = 84.39 (3.49); d2 = 98.00 (1.06)) and in d3 compared to Tertiary trials (Wilcoxon test: T = 10.00, p = 0.020, n = 12; mean % (SEM): Tertiary = 32.32 (5.82); d3 = 61.19 (6.97)).

### No-goal Trials

We found a significant difference among conditions in the percentage of trials in which subjects retrieve tools or objects (Friedman test: χ^2^ = 15.32, df = 3, p = 0.001; mean % trials (SEM): Primary = 52.59 (10.59), No-tools = 49.62 (8.17), No-food = 54.07 (9.06), Secondary-any = 79.99 (10.24)). Wilcoxon post-hoc test showed that subjects extracted tools more often in the Secondary-any trials than in the Primary condition (T = 10.00, p = 0.038, n = 11). The same was true for the No-tools (T = 4.00, p = 0.006, n = 12) and No-food conditions (T = 0.00, p = 0.001, n = 10). Subjects did not probe with the objects on food-platform. Only two subjects probed once (trials 2 and 5, respectively) when there was no food on the platform and one subject did so twice (trials 1 and 5).

## Discussion

All fifteen subjects targeted the out-of-reach tools in the Length-only trials and twelve out of fifteen subjects did so in the sequential trials. Subjects used up to 3 tools in a sequence. Subjects’ success in the sequential trials was determined by the complexity of the task; in fact, their performance was significantly diminished in the Tertiary trials, in which subjects were required to use 3 tools in a sequence to successfully retrieve the reward. They performed better in the Length-only trials than in the sequential trials only when the food was placed at the farthest distance from them.

Apes were able to recognize when they needed to use a tool to reach for another one because, in contrast to the Length-only or Primary trials, they used the longer within-reach tool in the sequential trials to reach for the out-of-reach tools first. Likewise, subjects’ performance in the Tertiary trials confirms these findings, because apes used 3 tools sequentially to get the reward. Subjects were able to solve the Secondary-any and Secondary-long conditions on the very first trial. However, this was not the case for the Tertiary condition, in which only four out of twelve subjects solved the problem in the first trial. Nevertheless, these results are remarkable since apes were neither trained in the different steps of the task nor received familiarization trials before the experiment.

Despite subjects’ good overall performance in the sequential trials, their correct responses in these trials were not perfect. In fact, “perfect” responses occurred more often in the Secondary-any and Secondary-long sequential trials than in the Tertiary trials; that is, in those trials in which the use of only 2 tools in sequence, rather than 3, was necessary to retrieve the food. Comparisons between sequential and Length-only trials confirmed that “perfect” performances occurred more often in d2 and d3 trials than in Secondary any and Tertiary trials, respectively; that is, when only the use of one tool was necessary to retrieve the food.

We examined no-goal trials to investigate whether tools were extracted only when required. Apes almost never probed with the tools when there was no food on the platform and they extracted more tools when it was required than when they were not needed for extractions (i.e. Primary, No-tools). Additionally, subjects never used the objects to retrieve the food from the platform. These results suggest that apes took into account the requirements of the different experimental situations and behaved accordingly. Note that even though in Wimpenny et al.’s study, crows probed the food-frame on fewer No-food trials than predicted by chance, all subjects did insert tools into the (empty) food-frame on at least one trial. Similar to our results, crows rarely probed for the food with the extracted non-tool objects.

Our results also showed that apes were sensitive to the distance of the food on the platform since they reached more often for the out-of-reach tools when the food was placed at a farther distance than when it was placed at a closer distance on the platform. Similar to Wimpenny et al’s results, we also found that when the food was positioned at an intermediate distance (d2), subjects tended to first try to retrieve the food with the longer within-reach tool. Wimpenny et al. suggested that these mistakes could be due to subjects’ difficulty at estimating how far they could reach with the longer within-reach tool. However, we believe that this explanation does not account for our results. Otherwise, subjects would have also used the shortest within-reach tool to reach for the food when it was placed at a closer distance. This was not the case. Therefore, we suggest that it is the short distance (less than 7 cm) between d1 and d2 that could have led our subjects to perceive both distances as being very similar and, in consequence, to use the 20 cm tool to reach for the reward in d2.

Similar to what it has been reported for crows, we also found that apes had a strong tendency to use first the longer out-of reach tools, irrespectively of condition. However, in those trials in which only the longest out-of-reach tool could be used to successfully retrieve the reward, apes only used the 55 cm tool more often in the Secondary-long condition but not in the Tertiary condition. Likewise, in the Length-only trials, apes selected the longest tool independently of the distance at which the food was placed. Although this may indicate that apes lack tool selectivity, it is conceivable that our setup was not adequate to detect it. In fact, except for the Tertiary condition, the out-of-reach tools were always all evenly aligned and, therefore, the costs of extracting any of the tools in those sequential trials were exactly the same. Interestingly, a closer inspection of the errors made in the correct trials helps to shed some light on this issue. Our results showed that in the Primary trials “perfect” performances occurred more often than in d1 trials. Subjects also made fewer errors in the Secondary-any than d2. We believe that these results could be due to the costs associated with the Primary and Secondary-any trials. When the longer tools were out of subjects’ reach, in the Primary condition apes tended to be more selective and use the 20 cm tool more often than when the longer tools were within subjects’ reach. The opposite is true for the Secondary-any trials: given that the longer tools were not within immediate reach, subjects tried to reach for the food with the 20 cm. We addressed this issue in the next experiment by introducing greater costs for retrieving the longer out-of-reach tools in the experimental conditions. If the apes were sensitive to these costs they should become more selective in their tool choices.

### Experiment 2

This experiment focused on tool selectivity when the costs of retrieving longer out-of-reach tools were increased. Indeed, previous research with humans [21], [22] and great apes [8] have shown that adding some type of cost (e.g. time to see the task) affects how selective subjects are at choosing tools with the appropriate length. In Experiment 2 we increased the costs of retrieving the longer out-of-reach tools by placing them at progressively longer distances from the mesh; so that only the shortest out-of-reach tool (25 cm) was reachable with the within-reach tool. Then, each tool had to be used sequentially in order to extract the next longer one. Thus, in order to obtain the longest out-of-reach tool, apes had to use 5 tools in a sequence. We predicted that if apes took into account the distance at which the food was placed on the platform, they should only retrieve the necessary number of tools according to the food depth and, consequently, only retrieve the longest out-of-reach tool when the food was placed at the farthest distance from them.

## Materials and Methods

### Subjects

We tested the same subjects that participated in Experiment 1.

### Apparatus

We used the same apparatus as in Experiment 1 except that we eliminated the use of corks and wooden bricks because subjects received no no-goal control trials.

### Procedure

We followed the same general procedure as in Experiment 1 with some changes. Whereas in Experiment 1 the food could be placed at 3 different distances, in Experiment 2 the reward was placed at 4 different distances (see Table 2). Thus, the sequence of behavior and the number of out-of-reach tools required to obtain the reward were dictated by the depth at which food was placed, with the most demanding condition requiring the use of 5 tools in a sequence (see Table 4 for an overview of all the conditions). Also, unlike Experiment 1, we excluded the No-tools or No-food control conditions, and we provided subjects with only one within-reach tool (20 cm) rather than two.

**Figure 4:**
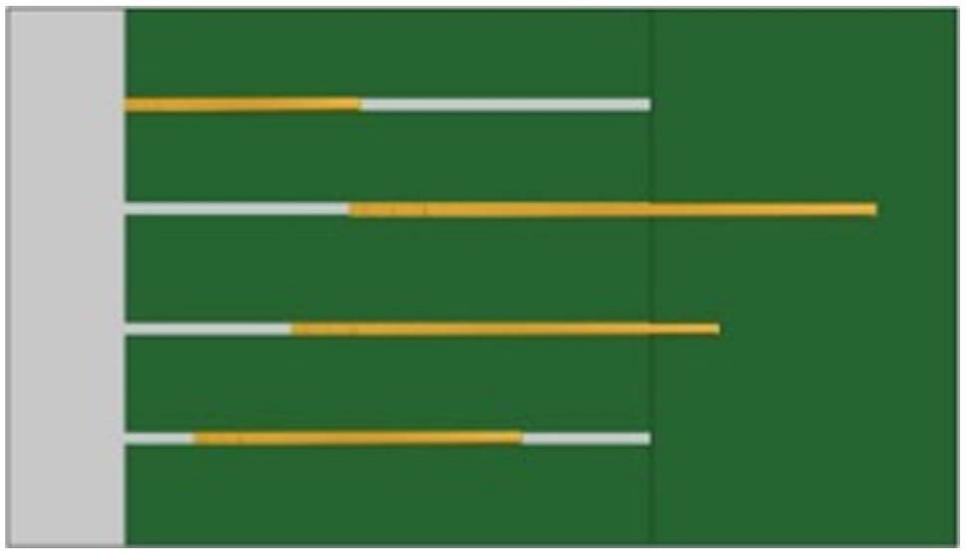
Illustration of the arrangement of the out-of-reach tools in Experiment 2 for the sequential trials (tools are shown in yellow).

All subjects received 2 types of trials (Table 5): sequential trials (Primary, Secondary, Quaternary and Quinary) and “Length-only” control trials (d1, d2, d3, d4). In contrast to Experiment 1, in the sequential trials the out-of-reach tools were not evenly aligned on the platform but were displaced by some distance one after the other with the shortest being the closest to the mesh and the longest being the farthest from the mesh (Figure 4). Thus for each type of sequential trial, only the 25 cm out-of-reach tool was directly retrievable with the 20 cm within-reach tool. Then, the 25 cm out-of-reach tool could be used to retrieve the 35 cm out-of-reach tool. Therefore, each out-of-reach tool had to be used to extract the next longer tool. Similar to Experiment 1, in the “Length-only” trials the tools were placed within subjects’ reach.

**Table 5:** Description of sequential and length-only conditions for Experiment 2.

Condition	Conditiontype	Foodposition	Trial description	Most task-sensitivebehavior for success
**Secondary-any**	Sequential	d2	The four out-of-reach tools are all displaced by some distance on the table. Food is reachable with any out-of-reach tool.	Get the 25 cm out-of-reach tool, only which is within reach of the 20 cm tool. Use the 25 cm tool to reach for the food.
**Quaternary**	Sequential	d3	The four out-of-reach tools are all displaced by some distance on the table. Food is only reachable with the second longest out-of-reach tool (45 cm).	Get the second longest out-of-reach tool (45 cm) with the 35 cm by extracting beforehand the 35 cm tool which is only reachable with the 25 cm tool which is only reachable with the 20 cm tool. Use the 45 cm tool to reach for the reward.
**Quinary**	Sequential	d4	The four out-of-reach tools are all displaced by some distance on the table. Food is only reachable with the longest out-of-reach tool (55 cm).	Get the longest out-of-reach tool (55 cm) with the 45 cm tool by extracting beforehand the 45 cm which is only reachable with the 35 cm which is only reachable with the 25 cm which is only reachable with the 20 cm. Use the 55 cm tool to reach for the food.
**Primary**	Control	d1	Food is within reach of the tool subjects are provided with (20 cm).	Reach for the food with the 20 cm. Do not probe for any out-of-reach tool.
**Length-only** **(d1/d2/d3/d4)**	Control	d1, d2, d3, d4	These four types of length-only trials correspond to the primary, secondary-any, quaternary and quinary trials. In contrast, the four tools are now placed on a tray within reach of the subjects.	Length-only (d1): reach for food with the 20 cm tool. Length-only (d2): Get any tool from the tray and then reach for the reward. Length-only (d3): Get the second longest tool (45 cm) from the tray and then reach for the food. Length-only (d4): Get the longest tool (55 cm) from the tray and reach for the reward.

Subjects received nine sessions of 8 trials each (72 trials in total). Each type of sequential and Length-only control trial were randomly assigned within one session and each type trial presented only once in each session. The position of the out-of-reach tools was counterbalanced across trials.

### Data Scoring and Analysis

We videotaped all trials. For each trial we coded the same responses that we coded in Experiment 1. A second independent observer scored a randomly selected sample of 20% of the trials to assess inter-observer reliability, which was excellent for all the variables (retrieved food: Cohen’s k = 1; within-reach tools: Cohen’s k = 0.97; first probing: Cohen’s k = 0.98; first out-reach tool taken: Cohen’s k = 0.97; first-out-of-reach tool used: Cohen’s k = 0.94). We coded subjects’ correct responses in the same way as in Experiment 1, that is, whether correct responses were “perfect” or whether they contained errors (see Table 5) for the expected perfect responses in relation to the food location and length of the tools).

We calculated the percentage of trials in which subjects obtained the reward (i.e. overall success). We used non-parametric tests because the data was not normally distributed. We used Friedman tests to analyze subjects’ success in the sequential trials and Length-only trials. Friedman tests were also used to investigate differences in first probing behaviors and first tools used. Wilcoxon tests were run for post-hoc comparisons and to assess whether subjects performed above chance levels. We used the Kruskal-Wallis test to investigate species differences. Mann-Whitney-U test was used to analyze whether order in which the two experiments were presented had any effect in chimpanzees’ performance. Exact p values were calculated in all cases. All tests were two-tailed.

## Results

We first checked whether chimpanzees’ performance was affected by the order in which they were presented with Experiment 1 and Experiment 2. A Mann-Whitney-U test revealed that success did not differ between the two groups of chimpanzees (Secondary-any: *U* = 7.500, *p* = 1.000; Quaternary: *U* = 7.000, *p* = 1.000; Quinary: *U* = 7.000, *p* = 0.857; Primary: *U* = 8.000, *p* = 1.000; *N* = 8 for all cases). Therefore, we pooled the data from the two groups for subsequent analyses.

### Sequential Trials

#### Success

**Figure 5:**
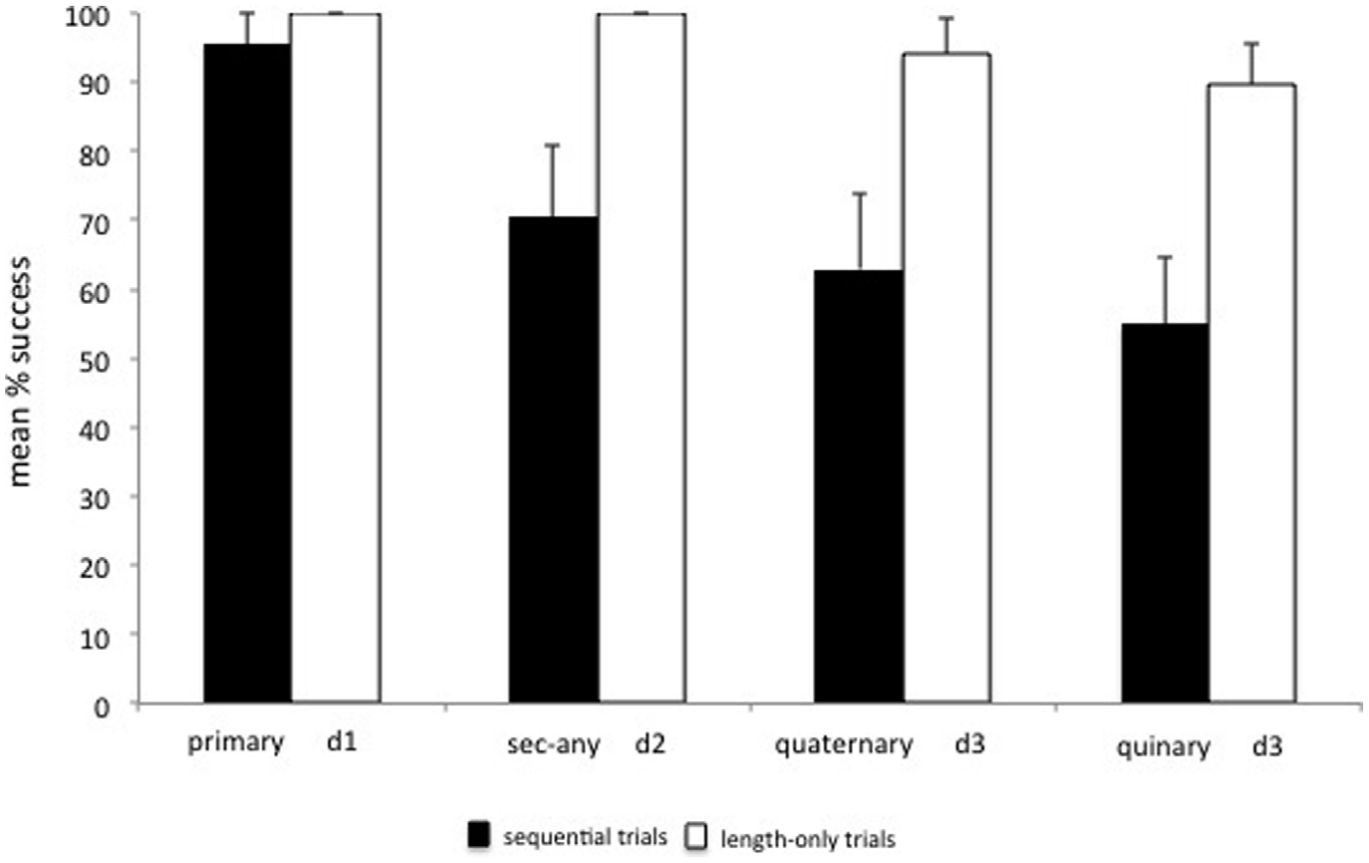
Mean % correct trials in the sequential and length-only trials (Experiment 2) [Error bars represent the standard error of mean].

Subjects’ overall success decreased as a function of task complexity (Friedman test: χ^2^ = 28.94, df = 3, p<0.001, Figure 5). Post-hoc tests showed that subjects performed significantly better in the Primary condition than in the Secondary-any (Wilcoxon test: T = 0.00, p = 0.016, n = 7), Quaternary (T = 0.00, p = 0.002, n = 10) and Quinary (T = 0.00, p<0.001, n = 10). Subjects also performed better in the Secondary-any than in Quaternary (T = 7.00, p = 0.039, n = 9) and the Quinary conditions (Wilcoxon test: T = 0.00, p = 0.002, n = 10).

There were no significant differences among species for any of the sequential trials (Kruskal-Wallis test: Primary: χ^2^ = 2.750, df = 2, *p* = 0.467; Secondary-any: χ^2^ = 3.356, df = 2, *p* = 0.193; Quaternary: χ^2^ = 4.164, df = 2, *p* = 0.216; Quinary: χ^2^ = 2.469, df = 2, *p* = 0.317; *N* = 15 in all cases). Subjects solved the Secondary-any (*χ^2^* = 7.14, df = 1, p = 0.008, 12/14 correct) significantly above chance in the first trial, but not the Quaternary (*χ^2^* = 3.00, df = 1, p = 0.083, 9/12 correct) or Quinary conditions (*χ^2^* = 1.33, df = 1, p = 0.248, 4/12 correct) (see Table 6 for individual performances).

**Table 6:** First session in which individuals solved each experimental condition in Experiment 2 and individual performances (%) in the sequential and length-only trials.

Subject		Session			Sequentialtrials			Length-		only		trials
	sec-any	quaternary	quinary	primary	sec-any	quaternary	quinary	d1	d2		d3	d4
Joey	3	-	-	100	11.11	0	0	100	100		88.89	66.67
Kuno	1	2	5	100	33.33	11.11	33.33	100	100		100	100
Yasa	1	1	1	100	77.77	88.89	77.78	100	100		100	88.89
Dokana	1	1	2	100	88.89	66.67	22.22	100	100		100	100
Padana	1	2	2	100	100	77.78	77.78	100	100		100	100
Pini	1	1	1	100	100	88.89	77.78	100	100		100	100
Bimbo	6	-	-	33.33	0	0	0	100	100		100	88.89
Alex	-	-	-	100	0	0	0	100	100		22.22	11.11
Fifi	1	1	1	100	100	100	100	100	100		100	100
Lome	1	1	3	100	100	100	77.78	100	100		100	100
Sandra	1	1	2	100	100	100	77.78	100	100		100	88.89
Alexandra	1	1	2	100	100	100	88.89	100	100		100	100
Jahaga	1	2	3	100	100	88.89	77.78	100	100		100	100
Frodo	1	1	1	100	44.44	22.22	22.22	100	100		100	100
Pia	1	1	2	100	100	100	88.89	100	100		100	100
**Median**	**1**	**1**	**2**									

#### Out-of-reach-tools or food

Except for 3 subjects (Joey, Alex and Bimbo), all subjects reached for the out-of-reach tools in the sequential trials (see Table 6 for individual performances in each type of trial). When we analyzed if subjects used the 20 cm tool to reach for food or to reach for the out-of-reach tools, we found significant differences in their performance among the 4 different types of trials (Friedman test: χ^2^ = 36.33 df = 3, p<0.001; (mean % trials (SEM): Primary = 21.62 (5.99), Secondary-any = 38.88 (7.79), Quaternary = 94.22 (2.64), Quinary = 96.75 (2.20)). Subjects reached for the out-of-reach tools more often in the Secondary-any (Wilcoxon test: T = 2.00, p = 0.021, n = 10), Quaternary (T = 3.00, p = 0.013, n = 14) and Quinary (T = 3.00, p = 0.013, n = 14) than in the Primary trials; they also reached for the out-of-reach tools more often in the Quaternary (T = 1.00, p = 0.003, n = 13) and Quinary (T = 1.00, p = 0.003, n = 13) than in the Secondary-any trials.

#### First out-of-reach tool

**Figure 6:**
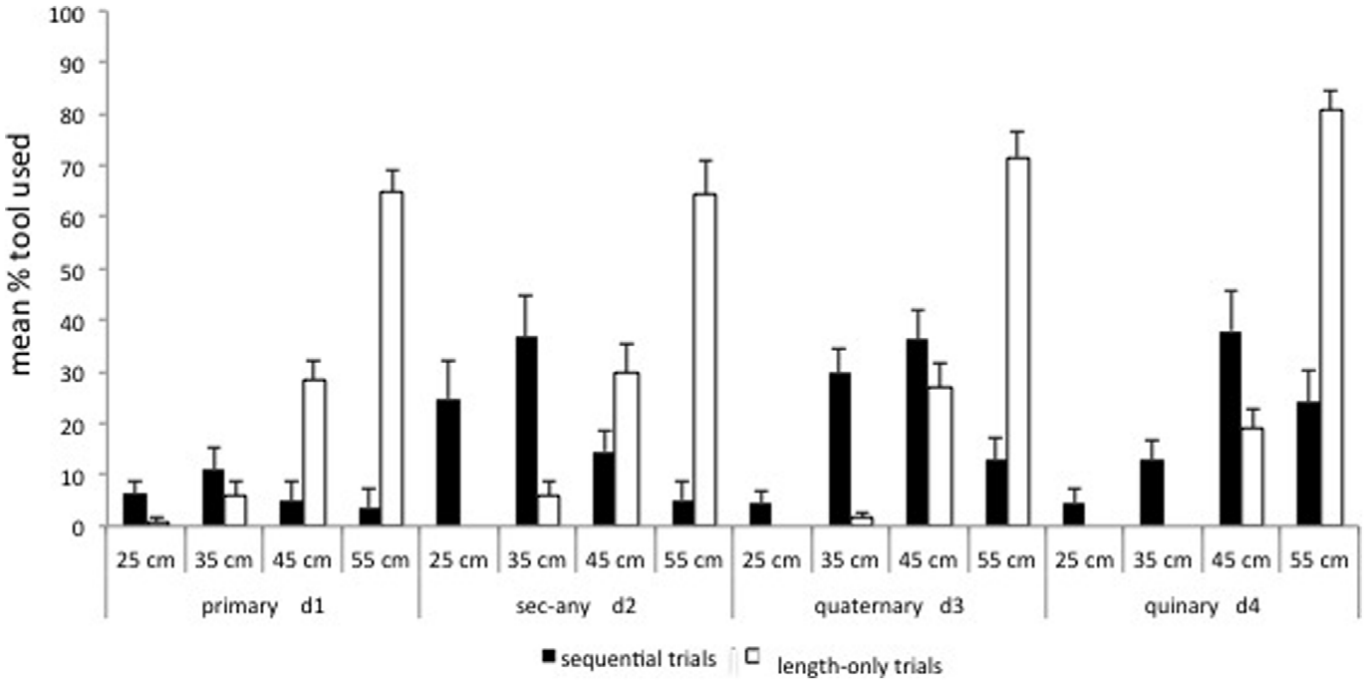
Mean % of first out-of-reach tool used in the sequential and length-only trials (Experiment 2) [Error bars represent the standard error of mean].

We found significant differences among the 4 out-of-reach tools that apes used to get the food in Secondary-any condition (Friedman test: χ^2^ = 9.75, df = 3, p = 0.017); Quaternary condition (Friedman test: χ^2^ = 18.90, df = 3, p<0.001), Quinary condition (Friedman test: χ^2^ = 10.89, df = 3, p = 0.012), but not in the Primary condition (Friedman test: χ^2^ = 2.14, df = 3, p = 0.693) (see Figure 6). Subjects had no clear preference for any of the more frequently used tools in the Secondary-any (Wilcoxon test: T = 26.50, p = 0.336, n = 12), Quaternary (Wilcoxon test: T = 24.00, p = 0.490, n = 11) or Quinary (Wilcoxon test: T = 20.50, p = 0.292, n = 11) conditions. In fact, when we analyzed if subjects were more selective in the Quinary condition (the only condition in which only the 55 cm tool could be used to retrieve the reward), we found that subjects did not significantly use the 55 cm out-of-reach first (Wilcoxon test: T = 21.50, p = 0.330, n = 11). A comparison of the average length of the tool selected in each condition revealed that tool length increased as a function of the distance to the reward (Friedman test: χ^2^ = 14.37, df = 3, p = 0.001, mean cm. (SEM): Primary = 32.77 (2.77), Secondary-any = 33.87 (1.74), Quaternary = 42.07 (1.03), Quinary = 44.97 (1.36)).

#### Errors

To further investigate subjects’ performance, we examined the errors that they made in the correct sequential trials. Results showed that subjects’ perfect responses in the Primary trials were more frequent that those containing errors (Wilcoxon test: T = 8.50, p = 0.002, n = 15; mean % (SEM): perfect = 81.47 (5.90); errors = 18.53 (5.90)). There were no differences between perfect and error performances in the Secondary-any trials (Wilcoxon test: T = 44.00, p = 0.946, n = 13; mean % (SEM): perfect = 51.04 (8.11); errors = 48.96 (8.11)), Quaternary (Wilcoxon test: T = 24.50, p = 0.789, n = 10; mean % (SEM): perfect = 52.54 (7.07); errors = 47.46 (7.07)) or Quinary (Wilcoxon test: T = 22.50, p = 0.375, n = 11, mean % (SEM): perfect = 40.98 (8.47); errors = 59.02 (8.47)).

### Length-only Trials

#### Success

Subjects’ overall success in these trials significantly differed among the 4 different types of Length-only trials (Friedman test: χ^2^ = 13.50, df = 3, p = 0.001). However, Wilcoxon post-hoc tests did not show significant differences between any of the conditions. When we compared subjects’ performance in the sequential tool trials with their corresponding trials in the Length-only condition, we found that subjects’ overall success was significantly better for the d2 condition than the Secondary-any condition (Wilcoxon test: T = 0.00, p = 0.016, n = 7) the d3 condition than the Quaternary condition (Wilcoxon test: T = 0.00, p = 0.002, n = 10) and d4 condition than Quinary condition (Wilcoxon test: T = 0.00, p<0.001, n = 14). Subjects only took the 20 cm in 1.11% of the trials.

#### First (out-of-reach) tool used

We found significant differences among the 4 tools that apes used to get the food in the d1 condition (Friedman test: χ^2^ = 38.88, df = 3, p<0.001); d2 (Friedman test: χ^2^ = 34.59, df = 3, p<0.001); d3 condition (Friedman test: χ^2^ = 40.80, df = 3, p<0.001) and d4 condition (Friedman test: χ^2^ = 42.75, df = 3, p<0.001). In all conditions, subjects showed a preference for using the longest out of reach tool (Wilcoxon test: d1 T = 0.00, p = 0.008, n = 9; d2: T = 3.00, p<0.001, n = 15; d3: T = 0.00, p<0.001, n = 15; d4: T = 0.00, p<0.001, n = 15) (see Figure 6). A comparison of the average length of the tool selected in each condition revealed a significant increase in tool length as a function of the distance to the reward (Friedman test: χ^2^ = 12.63, df = 3, p<0.004, mean cm. (SEM): d1 = 50.73 (0.66), d2 = 50.84 (0.82), d3 = 51.71 (0.67), d4 = 53.07 (0.36)).

#### Errors

Similar to the sequential trials, we also examined if in the correct trials, subjects’ performance was perfect or whether it contained errors. Subjects’ correct responses contained more errors than lack of them in d1 (Wilcoxon test: T = 0.00, p<0.001, n = 15; mean % (SEM): perfect = 1.48 (1.01); errors = 98.51 (1.01)) and d3 (Wilcoxon test: T = 3.50, p<0.001, n = 15; mean % (SEM): perfect = 21.57 (5.01); errors = 78.42 (5.01)). In contrast, subjects’ performance in d2 was always perfect. There were no significant differences between perfect performance and performance with errors in d4 trials (Wilcoxon test: T = 22.50, p = 0.375, n = 11; mean % (SEM): perfect = 54.71 (5.66); errors = 42.27 (5.66)). Next we compared perfect performances in the correct sequential trials and Length-only trials. We found that there were no significant differences in perfect performances between d4 and Quinary (Wilcoxon test: T = 27.50, p = 0.390, n = 12; mean % (SEM): d4 = 54.71 (5.66); Quinary = 40.98 (8.47)). Perfect responses occurred more often in d2 than Secondary-any (Wilcoxon test: T = 0.00, p<0.001, n = 12; mean % (SEM): d2 = 100.00 (0.00); Secondary-any = 51.04 (8.11)), between Quaternary than d3 (Wilcoxon test: T = 17.50, p = 0.070, n = 13; mean % (SEM): d3 = 21.57 (5.01); Quaternary = 52.54 (7.07)) and in Primary than d1 (Wilcoxon test: T = 0.00, p<0.001, n = 15; mean % (SEM): d1 = 1.48 (1.01); Primary = 81.47 (5.90)).

## Discussion

All fifteen subjects reached for the out-of-reach tools in the Length-only trials and thirteen out of fifteen subjects did so in the sequential trials. Subjects used up to 5 tools in a sequence. They performed better in the Length-only trials than in the sequential trials in those conditions in which the food was not reachable by the 20 cm within-reach tool. Similar to Experiment 1, subjects’ success in the sequential trials was determined by the complexity of the task; in fact, their performance was significantly diminished in the Quaternary and Quinary trials, in which subjects were required to use 4 and 5 tools, respectively, in a sequence to successfully retrieve the reward.

As in Experiment 1, we examined whether subjects attended to the position of the food by analyzing whether their first probe with the 20 cm tool was aimed at the out-of-reach tools or at the food. The results confirm our previous findings; that is, subjects used the immediately available tool to extract tools more often in all the sequential trials than in the Primary trials, in which the food was reachable with the 20 cm tool. Therefore, subjects were able to adjust their first probing actions to the distance of the reward and taking into account this information for further actions.

When we compared subjects’ performance in the sequential tool trials with their corresponding trials in the Length-only condition, we found that, except for subjects’ performance in d1 and Primary trials, subjects’ overall success was significantly better for all the Length-only trials than sequential trials. Thus, using tools in sequence imposed certain cognitive demands that resulted in a diminished performance compared to the Length-only trials. In fact, a closer look at subjects’ performance showed that perfect responses tended to occur when only one tool was required to obtain the reward (e.g. Primary trials). Similar to Experiment 1, subjects were able to solve the Secondary-any on the very first trial. It is also remarkable that nine out of twelve subjects solved the Quaternary task on the first trial and five subjects did so in the Quinary condition. These results are noteworthy since apes were not previously trained on the task.

Similar to Experiment 1, Experiment 2 demonstrated that apes were sensitive to the distance of the food on the platform since they reached more often for the out-of-reach tools when the food was placed at a farther distance than when it was placed at a closer distance. As in Experiment 1, we also found that when the food was positioned at an intermediate distance (d2), subjects tended to first try to retrieve the food with the immediate available tool. However, subjects reached for the out-of-reach tools more often in the Secondary-any condition than in the Primary condition. Thus, as the distance to the food increased, the likelihood of subjects first trying to reach for the out-of-reach tools increased.

Do apes use such information to select the appropriate out-of reach tool? We did find that subjects’ tool choices varied across the different experimental trials. In fact, our results showed that in the Secondary-any condition apes tended to choose more often the 25 cm and 35 cm tools than the longer out-of-reach tools (45 cm or 55 cm long tools). Likewise, they retrieved and used the 45 cm tool more often than any of the other out-of-reach tools in the Quaternary condition. Subjects used the 45 cm and 55 cm tools to try to reach for the food in Quinary condition; however they did not use the longest out-of-reach more often in this condition. In contrast, subjects’ performance in the Length-only trials followed a different pattern: apes selected the 45 cm and 55 cm long tools independently of the distance at which the food was placed. This finding confirms the results from Experiment 1.

In contrast to Experiment 1, in the sequential trials and Length-only trials we found a significant increase in tool length apes used as a function of the distance to the reward. This result is noteworthy for two reasons. First of all, it confirms that adding costs at retrieving the tools has an effect on tool selectivity. Second, even though there was no cost associated with choosing the 55 cm tool in the Length-only condition, we found that apes used such tool more often when the reward was farther away from them than when the reward was placed at a closer distance. One possibility is that the way in which the trials were presented affected subjects’ performance. Whereas in Experiment 1, we presented subjects with the sequential trials first and then with the Length-only trials, in Experiment 2 we intermixed both types of trials. Such procedural modification could have affected apes’ tool choices and facilitated more tool selectivity. Another possibility is that apes could potentially be selective even when there were no high costs involved at retrieving tools. However this is in contrast with the results from the sequential trials. Moreover, a closer look at the range of tools that apes used in both sequential and Length-only trials helps to shed light on this issue. Whereas in the Length-only trials apes’ choices mainly oscillated between the 45 cm and 55 cm tools, in the sequential trials apes were more selective depending on the distance at which the reward was placed; that is, they tended to use 25- and 35-cm tools when the food was closer to them and 45- and 55-cm tools when the reward was farther away.

A closer inspection at the errors made in the correct responses supports the idea that when reaching for tools is costly, apes become more selective. Similar to the results reported in Experiment 1, “perfect” performances occurred more often in the Primary trials than in d1 trials. In the Primary trials when the costs of reaching for the longer out-of-reach tools were increased, apes tended to be more selective and used the 20-cm tool more often than when the out-of-reach tools were within subjects’ reach. The opposite was true for the Secondary-any trials: when the longer out-of-reach tools were more costly to get, subjects tried to reach for the food more often with the 20 cm tool than with the out-of-reach tools. Additionally, our results showed that subjects made more mistakes in the Secondary-any than in d2 trials. Altogether these results provide strong support for the idea that increasing the costs associated with retrieving the tools significantly affects subjects’ tool selection responses. In other words, apes exhibited tool selectivity when not doing so was costly.

### General Discussion

Apes used up to 3 tools or 5 tools in sequence to obtain an out-of-reach piece of food. Subjects were able to solve the task requiring the use of two tools in sequence on the very first trial (Secondary-any and Secondary-long conditions) and adopted the use of more than two tools in sequence only after a few trials. Nevertheless, subjects’ performance was better when no sequential tool-use was required to get the food (i.e. Length-only trials) compared to when sequential tool use was required. Experiment 1 showed that subjects had a preference for using the longer out-of-reach tools even when a shorter tool sufficed to reach the reward. However, the results from Experiment 2 showed that increasing the costs of reaching for longest out-of-reach tool made apes more selective at choosing tools. So that their tool choices matched more closely the distance at which the food was located.

With regard to the aim of establishing comparisons across various species, our results confirm and extend previous findings on sequential tool-use in great apes [7], [8], [12]. Similar to Mulcahy et al.’s study [8], apes used tools sequentially in a spontaneous manner. This is in contrast with the study by Rensch & Dohl [12], in which the chimpanzee received several pre-training sessions before she was able to use 5 tools in sequence. In the sequential trials (Experiment 1) and Length-only trials (Experiment 1 & 2), apes showed an overall preference for the longer tools regardless of whether a shorter tool could also be used to get the reward. Similar to Mulcahy et al’s study [8], we also found that this preference changed when the costs of retrieving the longer out-of-reach tools were increased. In those trials in which the longer tool was unnecessary, apes tended to use either the short tool that was within reach or the shorter out-of-reach tools. We found no evidence of interspecific differences in sequential tool use even though bonobos, unlike chimpanzees and orangutans, do not regularly use tools in the wild. Mulcahy et al. [8] also found no differences between orangutans and gorillas, even though gorillas do not use tools in the wild.

Beyond primates, apes, just like New Caledonian crows, probed for the out-of-reach tools use on the first trial, even though none had been given previous training on the different steps of the problem. In contrast with the crows, however, apes were faster at solving the different types of trials. Whereas the crows solved the Secondary-any on the fourth and the Secondary-long on the fifth trial, apes did that on their very first trial. Even though our subjects did not solve the Tertiary condition significantly above chance on the first trial, they were able to solve it faster than the crows. In fact, most of the crows solved the Tertiary condition in an additional block of trials that they received after all the 54 trials were over (note that apes were not presented with this additional block of trials). Apes, like crows, directed more of their first probes towards tools when the food was further away, demonstrating that probing for tools was not simply a result of frustration at their inability to retrieve food. Similar to the crows, apes also showed a tendency for using longer out-of-reach tools in the sequential trials (Experiment 1). Apes, like crows, used longer tools to probe for food in Length-only, compared to Sequential trials.

Wimpenny et al. [1] argued that in order to qualify as goal-directed, tool-extraction should only occur when required (i.e. when the length of the within-reach tool is not long enough to reach for the food or when there is food on the platform). Wimpenny et al. [1] also argued that if probing for food was goal-directed, subjects should not probe on the food-platform neither in the Non-food nor the No-tools conditions. Since our subjects almost never probed with the tools when there was no food on the platform and they extracted more tools when it was required than when there was no need for tool extractions (i.e. Primary, No-tools), this suggests that apes exhibited goal-directed behavior. In contrast, Wimpenny et al. [1] found that even though crows showed flexibility in their behavior, by extracting tools on fewer trials when they were unnecessary, they still probed into the food-frame when food was absent, or could not be obtained.

Despite being able to use tools sequentially and successfully retrieve the reward, apes’ correct performance, just like crows’, was not perfect. In fact, subjects performed worse in the Sequential trials compared to the Length-only trials. Several reasons could explain this finding. One possibility is that the sequential condition taxed apes’ attentional resources. Whereas in the Length-only trials subjects could succeed by retrieving the longer tool, subjects in the Sequential trials not only had to recognize and respond to the depth of food, but they also had to decide whether to retrieve an additional tool. This may have divided their attention, a process that could have affected their performance [23]. Additionally, the extra effort required to obtain multiple tools in sequential trials compared to Length-only trials may have also contributed to the errors observed. However, this cannot be the whole explanation because errors also occurred in Length-only trials.

Another possibility is that apes could not encode and/or remember the precise distance at which the food was placed and relate it to the size of the tools that they could choose from. Instead subjects used a strategy based on selecting the longest tool available regardless of the distance at which the food was located [8], [22], [24]. This strategy has three main advantages. First, it insures success in every trial. Second, since all the out-of-reach tools were evenly aligned in all conditions except the Tertiary condition, it means that the costs of extracting any of the out-of-reach tools were exactly the same. Third, it bypasses the problem of having to encode the distance of the reward in relation to the lengths of the tools. Subjects could simply compare tools and select the longest one.

However, subjects’ responses in the Secondary-long and Tertiary trials of Experiment 1 do not fit the “select the longest tool available” rule. Instead, apes might have used a rule based on using the longest immediately available out-of-reach tool to get for the food; but if this is not successful then extract the next longest tool”. This would have allowed them to succeed by using the 55 cm tool in all but the Tertiary condition; and in the latter they would have first used the 45 cm tool since it was the longest immediately available tool. The failure at retrieving the food with the 45 cm tool would have forced them to retrieve the backwards-displaced 55 cm tool and use it to successfully reach for the food. Thus, this heuristic rule could explain subjects’ performance in Experiment 1. However, this rule cannot explain subjects’ responses in Experiment 2. More specifically, if apes were using such procedural rule, in the Quaternary condition they should have used the 35 cm tool more often than the 45 cm because such tool was the immediately available tool after the 25 cm. However this is not what we found.

Likewise, the results from Experiment 2 also indicated that subjects’ performance showed a different pattern depending on the costs involved in retrieving the out-of-reach tools: if the costs of reaching for the longest tool were increased, apes stopped extracting the longer out-of-reach tools when a shorter one suited the task requirements. That is, presenting apes with a more demanding task revealed that subjects were indeed capable of encoding the distance to the reward in relation to the length of the tools available. In general, these results mirror those showing that making a task more demanding can, in some cases, contribute to uncover abilities thought to be beyond apes’ grasp [25], [26].

Altogether the results reported here demonstrate that apes were able to perform multiple steps of a complex behavioral sequence and, consequently, act in a goal-directed manner by using a tool to access as many out-of-reach tools as necessary in order to get a reward. Likewise, differences in performance in the different types of sequential trials together with subjects’ better performance in the length only trials also indicate that the complexity and cognitive demands (e.g. level of anticipatory planning or hierarchically organized behavior) involved in sequential tool use, increased with the number of steps necessary to achieve the final outcome. Such finding is also congruent with the Wimpenny et al. [1]’s results in their equivalent experiment with New Caledonian crows. The methodological extension presented here also sheds light on tool selectivity in great apes. Increasing the cost of reaching for the longest tool showed that apes could be selective at choosing tools. The findings of the present studies also contribute to the growing body of research revealing great apes also use tools to act upon another object. Extending these findings (Experiment 2) to other species (e.g. New Caledonian crows) would also be crucial to understand not only which mechanisms drive tool-use behavior but also under which conditions tool-use behavior evolved.
